# Association between serum uric acid and arterial stiffness in a large‐aged 40–70 years old population

**DOI:** 10.1111/jch.14527

**Published:** 2022-06-24

**Authors:** Alexandre Vallée

**Affiliations:** ^1^ Department of Epidemiology‐Data‐Biostatistics Delegation of Clinical Research and Innovation (DRCI) Foch hospital Suresnes France

**Keywords:** arterial stiffness, arterial stiffness index, atherosclerosis, cardiovascular disease, sex difference, urate, uric acid

## Abstract

Arterial stiffness (AS), measured by arterial stiffness index (ASI), is a determinant in cardiovascular (CV) diseases. A high serum uric acid (SUA) level is a known risk factor for CV disease. The authors investigated the relationship between SUA and ASI in the middle‐age UK Biobank population study. AS was defined as ASI > 10 m/s. A cross‐sectional study was conducted from 126 663 participants. Participants were divided into four quartiles according to SUA levels and sex. Sex multivariate analyses were performed with adjustment for confounding factors. The average ASI for overall participants was 9.3 m/s (SD: 2.9); 9.9 m/s (SD: 2.8) for men and 8.7 m/s (SD: 2.9) for women (*P* < .001). Men presented higher SUA rate (351.3 mmol/L (SD:67.9)) than women (270.7 mmol/L (SD:64.4)), *P* < .001. In men multivariate analysis, SUA remained a determinant of AS, with an increase in the strength of the association between the quartiles, Q4 versus Q1, OR = 1.10 [1.05–1.16], *P* < .001, Q3 versus Q1, OR = 1.09 [1.04–1.14], *P* < .001 but not between Q2 and Q1 (*P* = .136). In women, SUA remained significant for AS, with an increase in the strength of the association between the quartiles, Q4 versus Q1, OR = 1.22 [1.15–1.30], *P* < .001, Q3 versus Q1, OR = 1.13 [1.07–1.19], *P* < .001 and no difference between Q2 and Q1 (*P* = .101). When applying continuous SUA values in the multivariate analysis, SUA remained significant (*P* < .001), with a Youden index value for men = 338.3 mmol/L and for women = 267.3 mmol/L. High SUA levels were associated with AS, suggesting that SUA could be used as a predictor of atherosclerosis.

## 1 INTRODUCTION

Atherosclerosis is a main pathological mechanism of cardiovascular (CV) diseases,^1^ but early atherosclerosis is lack of specific manifestations, thus, atherosclerosis is easy to be ignored in its early stages.^2^ Therefore, how to identify arterial stiffness early is an important strategy to prevent and manage CV diseases. With the gradual development of medical equipment and the continuous improvement of people's attention to CV diseases, the technology of evaluating arterial stiffness by non‐invasive method has become the focus of clinicians.

Arterial stiffness, measured by arterial stiffness index (ASI), can be considered as a major denominator in target organ damage.^3^ Numerous noninvasive arterial parameters have been shown to be biomarkers of arterial stiffness.^4^ Arterial stiffness is the arteries capacities to expand and contracts face to the cardiac flow. Arterial stiffness can be an integrator of long‐lasting arterial wall damage leading to a luminal dilation due to increase in collagen deposition.^5^ Arterial stiffness is associated with coronary atherosclerosis,^6^ CV events^7^ or inflammatory disorders.^8^

Several studies have shown that carotid‐femoral (aortic) pulse wave velocity (PWV) can considered as the criterion standard of arterial stiffness. PWV levels are strongly correlated with risk factors such as atherosclerosis,^9^ hypertension and diabetes,^10^ and CV diseases.^11^ Nevertheless, PWV measurement is time‐consuming and operator dependent.

In parallel, ASI is a simple, operator independent, convenient and noninvasive device to measure arterial stiffness through the utilization of infrared light (photoplethysmography) to record the volume waveform of the blood into the finger. The shape of the waveform is directly associated to the time it takes for the pulse wave to cross by the arterial tree. These ASI tools could be of interest for rapid estimation of CV risk.^5^, ^12^ A high serum uric acid (SUA) level is a known risk factor for cardiovascular disease.^13^, ^14^ Epidemiologic studies have also found an association between high SUA level and arterial stiffness in patients with various disorders such as diabetes, hypertension, and chronic kidney disease.^15^ But this relationship remains still controversial and few reports were performed in general population.^14^, ^16^, ^17^

Thus, our cross‐sectional study investigated the association between SUA and arterial stiffness as evaluated by ASI in the large middle‐aged UK Biobank population.

## 2 METHODS

### 2.1 UK Biobank population

The UK Biobank is a prospective cohort for the investigation, prevention, diagnosis and treatment of chronic diseases, such as CV diseases in adults. 502 478 Britons aged 40 to 70 years old across 22 UK cities from the UK National Health Service Register were included between 2006 and 2010. The cohort was phenotyped and genotyped, by participants who responded to a questionnaire; a computer‐assisted interview; physical and functional measures; and blood, urine, and saliva samples.^18^ Data included socio‐economic, behavior and lifestyle, mental health battery, clinical diagnoses and therapies, genetics, imaging and physiological biomarkers from blood and urine samples. The cohort protocol can be found in literature.^19^ All participants provided electronic informed consent and UK Biobank received ethical approval from the North‐West Multi‐center Research Ethics Committee (MREC) covering the whole of UK. The study was conducted according to the guidelines of the Declaration of Helsinki, and approved by the North West – Haydock Research Ethics Committee (protocol code: 21/NW/0157, date of approval: 21 June 2021). For details https://www.ukbiobank.ac.uk/learn‐more‐about‐uk‐biobank/about‐us/ethics

### 2.2 Blood pressure measurement

Systolic and diastolic blood pressure (SBD, DBP) were measured twice at the assessment center by the use of an automated BP device (Omron 705 IT electronic blood pressure monitor; OMRON Healthcare Europe B.V. Kruisweg 577 2132 NA Hoofddorp), or manually by the use of a sphygmomanometer with an inflatable cuff in association with a stethoscope if the blood pressure device failed to measure the BP or if the largest inflatable cuff of the device did not fit around the individual's arm.^20^

The participant was sitting in a chair for performing all the measures. The measures were carried out by nurses trained in performing BP measures.^21^ Multiple available measures for one participant were averaged. The Omron 705 IT BP monitor has satisfied the Association for the Advancement of Medical Instrumentation SP10 standard and was validated by the British Hypertension Society protocol, with an overall “A” grade for both SBP and DBP.^22^ Nevertheless, automated devices measure higher BP in comparison to manual sphygmomanometers, thus, we adjusted both SBP and DBP which were measured using the automated device using algorithms^23^:

For SBP, we performed the following algorithm:

$$\begin{array}{ccc} SBP\; & = & 3.3171+0.92019\times \;SBP\;\left(mmHg\right)+6.02468\\ & & \times \;sex\;\left(male\;=\;1;\;female\;=\;0\right) \end{array}$$

For DBP, we performed the following algorithm:

$$\begin{array}{ccc} DBP\; & = & 14.5647+0.80929\times \;DBP\;\left(mmHg\right)+2.01089\\ & & \times \;sex\;\left(male\;=\;1;female\;=\;0\right) \end{array}$$

These adjusted BP values were used for all calculations, including mean BP calculation.

Mean BP was calculated as:

$$mean\;BP\;=\frac{\left(SBP+2\times DBP\right)}{3}\;$$

### 2.3 Outcomes

Pulse wave arterial stiffness index (ASI) was measured by non‐invasive method during a volunteer's visit to a UK Biobank Assessment Center. Pulse waveform was taken by clipping a photoplethysmograph transducer (PulseTrace PCA 2, CareFusion, USA) to the rested volunteer's finger (any finger or thumb, mainly the index finger). Volunteers were asked to breathe in and out slowly five times in a relaxed fashion and readings were taken over a 10–15 s time. ASI is performed from a single peripheral pulse waveform. The carotid‐to‐femoral pulse transit time was estimated from the dicrotic waveform as the time difference between a forward compound when the pressure is transmitted from the left ventricle to the finger and a reflected or backward compound as the wave is transmitted from the heart to lower body via the aorta.^24^ ASI was estimated in meters per second (m/s) as: H/PTT. H is the individual's height, and PTT is the pulse transit time or the peak‐to‐peak time between the systolic and diastolic wave peaks in the dicrotic waveform.^24^ This methodology has been validated by comparing it with carotid‐femoral PWV. These studies concluded that both measure methods were highly correlated. ASI was a simple, operator independent, non‐expensive and rapid method.^12^, ^25^, ^26^ We excluded extreme outlier ASI values from the analyses. ASI > 10 m/s defined arterial stiffness.

### 2.4 Laboratory and clinical parameters

Hypertension was defined as systolic blood pressure (SBP) at least 140 mm Hg and/or diastolic BP (DBP) at least 90 mm Hg, according to guidelines by the European Society of Cardiology, and/ or antihypertensive drug used^27^ or hypertension diagnosed by a doctor. Diabetes status was defined on either receiving anti‐diabetic medication or diabetes diagnosed by a doctor or a fasting glucose concentration ≥7 mmol/L. Dyslipidemia was defined as having a fasting plasma total‐cholesterol or triglycerides level of ≥6.61 mmol/L (255 mg/dl) or > 1.7 mmol/L (150 mg/dl), respectively, or having statins medication.^28^ Calculated‐glomerular filtration rate (GFR) (by MDRD formula, MDRD: modification of diet in renal disease, by ml/min/1.73m^2^; GFR < 60 ml/min/1.73 m^2^ defined chronic kidney disease (CKD)). Current tobacco smokers were defined as participants who responded “yes, on most or all days” at the question “do you smoke tobacco now”. CV diseases were defined by heart attack, angina and stroke, as diagnosed by a doctor and reported in questionnaires. Medications were characterized by the question: “Do you regularly take any of the following medications?”. Uric acid lowering therapy was defined as reported by patients who had allopurinol, benzbromarone, febuxostat, probenecid or sulfinpyrazone.^29^ Body mass index was calculated as weight (in kg) divided by heigh^2^ (meter). ASI > 10 m/s defined arterial stiffness. Biological parameters were detailed in the UK Biobank protocol.^30^

### 2.5 Study population

Based on the 502 478 volunteers of the UK Biobank, participants with missing data and extreme values of ASI (defined as mean +/‐ 5*standard deviation)^31^ and extreme values of SUA (.5% participants with extreme values)^32^ were excluded to include 126 663 participants for the analyses.

### 2.6 Statistical analysis

Characteristics of the study population were described as the means with standard deviation (SD) for continuous variables and number and percentage for categorical variables. Analyses were performed via a sex stratification. SUA levels were categorized into quartiles.^16^, ^33^ To compare characteristics among the quartiles, we used the one‐way ANOVA test for continuous variables and the chi‐square test for categorical variables.

Arterial stiffness was defined as ASI > 10 m/s. Multivariate logistic analyses were performed to assess the relationship between arterial stiffness and SUA according to sex and with adjustment for BMI, GFR, C reactive protein (CRP), heart rate (HR), mean BP, age, testosterone, Insulin‐like Growth Factor One (IGF‐1), high density lipoprotein (HDL) cholesterol, fasting glucose, total cholesterol, triglycerides, tobacco status, low density lipoprotein (LDL) cholesterol, creatinine, urea, uric acid lowering therapy and CV diseases (+hormone therapy for women). Mean BP was determined for adjustment factor due to the high collinearity observed between SBP, DBP and MBP. Each parameter was performed independently to assess its association with arterial stiffness, SBP showed an AUC = .605, DBP an AUC = .611 and mean BP an AUC = .616, thus, mean BP was included in the multiple regression models because of its higher association with arterial stiffness.

Associations between SUA levels and ASI values were examined with linear regression models with adjustment for all confounding factors. Interactions were examined by including simultaneously cannabis use status and one of the covariates, their interaction term and adjustment for all other covariates.

The maximum Youden index of SUA values, performed as:

$$J\;=max_{c}\;\left[S_{e}\left(c\right)+S_{p}\left(c\right)-1\right]$$

was chosen to determine the optimal decision thresholds (*c*) for the discrimination based on $S_{e}$ as sensitivity and $S_{p}$ as specificity.^34^

A sensitivity analysis was performed by creating two subgroups of population: a population without CV diseases, diabetes and hypertension (*N* = 61 453) and a population with at least a CV disease, diabetes or hypertension (*N* = 65 210). Multivariate logistic analyses were performed to assess the relationship between arterial stiffness and SUA according to sex and these two groups with adjustment for all covariates.

Statistics were performed using SAS software (version 9.4; SAS Institute, Carry, NC, USA). A *P* value < .05 was considered statistically significant.

## 3 RESULTS

The clinical and biological characteristics of our population were stratified into four quartiles groups according to the distribution of the SUA values (**Table** **1**). The four quartiles of SUA were defined as follows: for men, SUA < 303.9 mmol/L for Q1, Q2: 303.9 mmol/L to 348.1 mmol/L, Q3/ 349.1 mmol/L to 396.1 mmol/L and Q4 > 396.1 mmol/; for women, Q1: SUA < 225.4 mmol/L, Q2: 225.4 mmol/L to 264.3 mmol/M, Q3: 264.3 mmol/L to 309.2 mmol/L, and for Q4 > 309.2 mmol/L.

**TABLE 1:** Baseline characteristics of total study population by quartiles (*n* = 126 663)

	Q1		Q2		Q3		Q4		
MEN	*N* = 16 038		*N* = 16 046		*N* = 16 009		*N* = 15 999		*P* value
Hypertension	8120	50.6%	8765	54.6%	9548	59.6%	11040	69.0%	<.001
Diabetes	1827	11.4%	1237	7.7%	1176	7.3%	1355	8.5%	<.001
Dyslipidemia	8683	54.1%	9823	61.2%	10757	67.2%	12098	75.6%	<.001
Chronic Kidney disease (CKD)	117	.7%	154	1.0%	285	1.8%	693	4.3%	<.001
Antihypertensive medication	3304	20.8%	3426	21.5%	3901	24.6%	5467	34.5%	<.001
Anti‐diabetes medication	1183	7.4%	706	4.4%	639	4.0%	722	4.5%	<.001
Statins	3682	23.2%	3572	22.5%	3818	24.0%	4515	28.5%	<.001
Hormone medication	–	–	–	–	–	–	–	–	–
CV diseases	1177	7.3%	1160	7.2%	1255	7.8%	1522	9.5%	<.001
Current tobacco	1764	11.0%	1403	8.7%	1224	7.6%	1010	6.3%	<.001
Arterial Stiffness Index (ASI), m/s	9.71	2.85	9.85	2.88	9.99	2.80	10.13	2.80	<.001
ASI > 10 m/s	7108	44.3%	7439	46.4%	7832	48.9%	8117	50.7%	<.001
Age (years)	56.89	8.29	56.88	8.32	57.06	8.25	57.21	8.19	<.001
BMI (kg/m^2^)	26.22	3.89	27.24	3.85	28.18	3.99	29.55	4.35	<.001
SBP mm Hg	136.72	15.99	138.00	15.85	139.10	15.85	140.63	15.82	<.001
DBP mm Hg	82.92	7.86	83.94	7.85	84.84	7.94	85.84	8.02	<.001
MBP mm Hg	100.85	9.78	101.96	9.70	102.93	9.71	104.10	9.68	<.001
HR bpm	66.61	11.39	66.75	11.29	67.61	11.57	68.91	12.12	<.001
C reactive protein (CRP), mg/L	2.13	4.42	2.20	4.14	2.36	3.85	2.85	4.30	<.001
Glucose, mmol/L	5.37	1.80	5.20	1.20	5.19	1.05	5.25	1.02	<.001
HbA1c, mmol/L	37.24	9.68	36.28	7.07	36.23	6.17	36.63	6.63	<.001
HDL cholesterol, mmol/L	1.34	.33	1.30	.31	1.28	.30	1.25	.30	<.001
LDL cholesterol, mmol/L	3.35	.83	3.46	.84	3.52	.87	3.52	.89	<.001
Total cholesterol, mmol/L	5.33	1.08	5.46	1.09	5.53	1.13	5.54	1.18	<.001
Triglycerides, mmol/L	1.65	.94	1.83	1.02	1.98	1.10	2.25	1.26	<.001
Testosterone, nmol/L	12.88	3.98	12.32	3.75	11.89	3.55	11.22	3.47	<.001
Insulin like Growth Factor (IGF), nmol/L	22.16	5.66	22.29	5.47	21.90	5.41	21.38	5.50	<.001
Serum Uric Acid (SUA), mmol/L	274.5	38.6	326.9	21.6	370.7	23.8	432.2	50.1	<.001
Uric acid lowering therapy (*n*, %)	557	3.47%	334	2.08%	300	1.87%	337	2.11%	<.001
Glomerular filtration rate (GFR), ml/min/1.73 m^2^	99.24	18.95	94.14	16.31	90.87	16.14	86.83	16.67	<.001
Creatinine, micromol/L	77.11	16.85	80.35	12.00	83.13	17.66	86.98	19.00	<.001
Urea, mmol/L	5.29	1.30	5.46	1.25	5.61	1.32	5.87	1.56	<.001

	Q1		Q2		Q3		Q4		
WOMEN	*N* = 15 660		*N* = 15 641		*N* = 15 654		*N* = 15 616		*P* value
Hypertension	4104	26.2%	5021	32.1%	6250	39.9%	8896	57.0%	<.001
Diabetes	549	3.5%	535	3.4%	744	4.8%	1417	9.1%	<.001
Dyslipidemia	5637	36.0%	6903	44.1%	8336	53.3%	10583	67.8%	<.001
Chronic Kidney disease (CKD)	58	.4%	115	.7%	305	1.9%	914	5.9%	<.001
Antihypertensive medication	1312	8.4%	1781	11.5%	2589	16.6%	5134	33.0%	<.001
Anti‐diabetes medication	284	1.8%	265	1.7%	343	2.2%	704	4.5%	<.001
Statins	1178	7.6%	1412	9.1%	1958	12.6%	3123	20.1%	<.001
Hormone medication	969	6.2%	1055	6.8%	995	6.4%	927	6.0%	.026
CV diseases	318	2.0%	351	2.2%	475	3.0%	783	5.0%	<.001
Current	1140	7.3%	1017	6.5%	984	6.3%	929	5.9%	<.001
Arterial Stiffness Index (ASI), m/s	8.26	2.80	9.48	2.91	8.78	3.01	9.27	3.15	
Arterial Stiffness (ASI > 10 m/s)	3801	24.3%	4265	27.3%	4916	31.4%	5848	37.4%	<.001
Age (years)	54.00	8.31	55.36	8.21	56.68	7.96	58.31	7.43	<.001
BMI (kg/m^2^)	24.70	3.84	26.04	4.31	27.55	4.77	30.45	5.84	<.001
Systolic BP mm Hg	123.66	17.29	126.45	17.53	128.95	17.54	132.48	17.27	<.001
Diastolic BP mm Hg	78.00	7.80	79.37	7.84	80.49	7.93	82.17	7.94	<.001
Mean BP mm Hg	93.22	10.17	95.06	10.21	96.64	10.20	98.94	9.92	<.001
HR bpm	68.38	9.76	68.71	10.02	69.47	10.46	71.10	11.29	<.001
C reactive protein (CRP), mg/L	1.73	3.53	2.09	3.53	2.63	3.88	3.91	4.80	<.001
Glucose, mmol/L	5.05	1.04	5.07	.89	5.13	.93	5.28	1.03	<.001
HbA1c, mmol/L	34.87	6.06	35.21	5.52	35.89	5.64	37.49	6.78	<.001
HDL cholesterol, mmol/L	1.68	.37	1.65	.37	1.60	.38	1.50	.37	<.001
LDL cholesterol, mmol/L	3.50	.82	3.60	.84	3.69	.87	3.74	.92	<.001
Total cholesterol, mmol/L	5.76	1.07	5.87	1.09	5.96	1.13	5.97	1.20	<.001
Triglycerides, mmol/L	1.25	.63	1.39	.71	1.56	.80	1.88	.97	<.001
Testosterone, nmol/L	1.08	.60	1.10	.57	1.12	.70	1.18	.73	<.001
Insulin like Growth Factor (IGF), nmol/L	22.10	5.73	21.67	5.73	21.15	5.68	19.94	5.62	<.001
Serum Uric Acid (SUA), mmol/L	200.6	32.4	245.4	19	284.9	21.8	344.9	52	<.001
Uric acid lowering therapy (n, %)	26	.17%	20	.13%	30	.19%	33	.21%	.321
Glomerular filtration rate (GFR), ml/min/1.73 m^2^	98.22	16.67	92.77	15.64	88.98	15.88	84.32	16.92	<.001
Creatinine, micromol/L	60.32	9.41	63.22	13.29	65.37	13.21	68.68	14.06	<.001
Urea, mmol/L	4.72	1.11	4.99	1.14	5.23	1.19	5.62	1.45	<.001

Categorical values: *n* and %, continuous values: mean and SD (standard deviation), SUA: median and interquartile range [IQR].

Abbreviations: CV, cardiovascular; BP, blood pressure; BMI, body mass index.

Sixty four thousand and nine two (50.6%) of the participants were men and 62 571 (49.4%) were women. Mean age of the overall participants was 56.6 years (SD: 8.2, minimal age: 40 years and maximal: 70 years). The mean average ASI for all the participants was 9.3 m/s (SD: 2.9), 9.9 m/s (SD: 2.8) for men and 8.7 m/s (SD: 2.9) for women (men vs. women, *P* < .001). Men presented higher rate of SUA (351.3 mmol/L (SD:67.9)) than women (270.7 mmol/L (SD:64.4)), *P* < .001. Only 1.29% of the overall population had uric acid lowering therapy, with a higher rate in men than in women (2.38% vs. .17%, *P* < .001). Only 7.5% of our study population was current smoker, but this rate was higher in men than in women (8.4% vs. 6.5%, *P* < .001).

**FIGURE 1:**
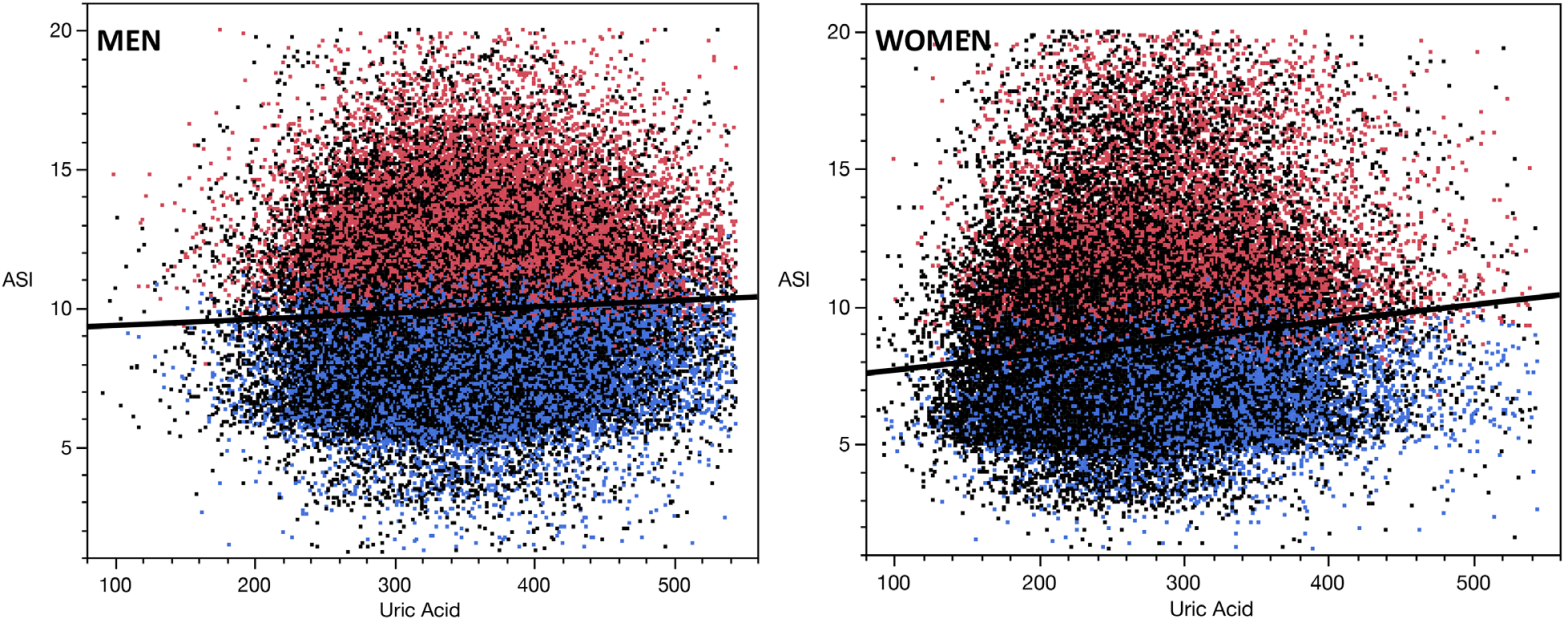
Linear relationship between SUA and ASI. SUA: serum uric acid; ASI: arterial stiffness index

A linear relationship between SUA levels and ASI values was observed among men (*P* < .001) and women (*P* < .001) (**Figure** **1**) which remained significant after adjustment for all confounding factors, among men (*P* = .003) and women (*P* < .001).

**FIGURE 2:**
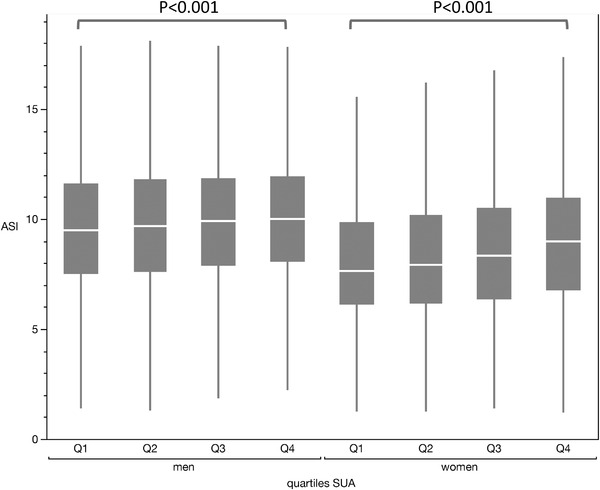
ASI values according to SUA quartiles and sex

**FIGURE 3:**
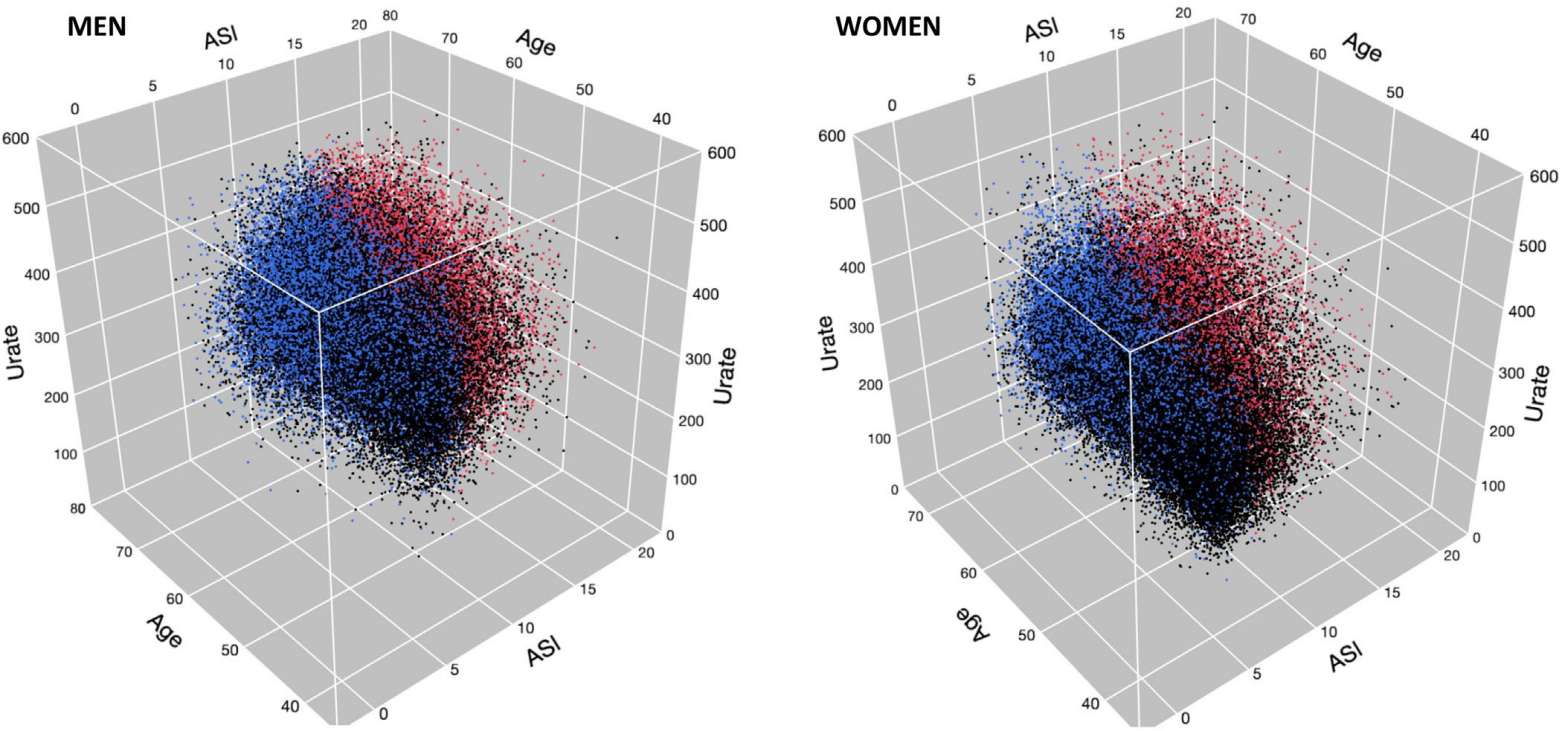
3D representation of the interaction between age and SUA on ASI values

A significant increase was observed for ASI levels across the different quartiles of SUA levels in both sex (*P* < .001) (**Table** **1**, **Figure** **2**), and this with an age‐interaction with SUA for ASI levels (in both sex, p for interaction < .001) (**Figure** **3**).

Those in the higher quartile of SUA showed higher hypertension, diabetes, dyslipidemia, CKD, CV diseases in both sex (*P* < .001, for all covariates) (**Table** **1**). Moreover, participants in the higher quartiles presented higher mean average of age, BMI, blood pressure, heart rate, CRP, fasting glucose, LDL cholesterol, total cholesterol, triglycerides, IGF‐1 than the others for both sex (*P* < .001), but lower HDL cholesterol and GFR levels. In contrast, men in the higher quartiles showed lower mean average of testosterone than the other quartiles (11.2 nmol/L (3.5) for Q4 vs. 12.9 nmol/L (4.0) for Q1), whereas it was the inverse among women (1.2 nmol/L (.7) for Q4 vs. 1.1 nmol/L (.6) for Q1, *P* < .001).

In the multivariate analysis among men, after adjustment for confounding factors, SUA remained an independent factor of arterial stiffness (defined by ASI > 10 m/s), with an increase in the strength of the association between the quartiles, that is, Q4 versus Q1, OR = 1.10 [1.05–1.16], *P* < .001, Q3 versus Q1, OR = 1.09 [1.04–‐1.14], *P* < .001 and no difference between Q2 and Q1 (*P* = .136). In the multivariate analysis, BMI (*P* < .001), HR (*P* = .048), Mean BP (*P* < .001), age (*P* < .001), IGF (*P* < .001), HDL cholesterol (*P* < .001), fasting glucose (*P* < .001), total cholesterol (*P* < .001), triglycerides (*P* < .001), tobacco status (*P* < .001), creatinine (*P* = .045), CV diseases (*P* = .006) and uric acid lowering therapy (*P* = .032) remained significant (**Table** **2**). When applying continuous SUA values in the multivariate analysis, SUA remained significant (*P* < .001), with a Youden index value equal to 338.3 mmol/L.

**TABLE 2:** Multivariate logistic analyses for arterial stiffness (ASI > 10 m/s) for men, women and overall study population

Men		
Parameters	OR 95%CI	P value
BMI (kg/m^2^)	1.02 [1.01–‐1.03]	<.001
Glomerular filtration rate (GFR), ml/min/1.73 m^2^	1.00 [.99–1.01]	.106
C reactive protein (CRP), mg/L	1.00 [.99–1.01]	.335
HR bpm	1.01 [1.00–1.02]	.048
Mean BP mm Hg	1.02 [1.01–1.03]	<.001
Age, years	1.06 [1.05–1.07]	<.001
Testosterone, nmol/L	1.00 [.99–1.01]	.379
Insulin like growth factor (IGF), nmol/L	.98 [.97–.99]	<.001
HDL cholesterol, mmol/L	.71 [.62–.80]	<.001
Glucose, mmol/L	.97 [.96–.98]	<.001
Total cholesterol, mmol/L	1.03 [.92–1.16]	.573
Triglycerides, mmol/L	1.07 [1.04–1.10]	<.001
Current tobacco (yes)	1.64 [1.54–1.74]	<.001
CV diseases (yes)	1.09 [1.03–1.16]	.006
Creatinine, micromol/L	.99 [.98–1.00]	.045
LDL cholesterol, mmol/L	1.01 [.88–1.16]	.926
Uric acid lowering therapy	.89 [.80–.99]	.032
Urea, mmol/L	1.00 [.99–1.01]	.436
Serum Uric Acid (SUA), mmol/L		<.001
°Q1	Ref.	
°Q2	1.04 [.99–1.09]	.136
°Q3	1.09 [1.04–1.14]	<.001
°Q4	1.10 [1.05–1.16]	<.001

Women		
Parameters	OR 95%CI	P value
BMI (kg/m^2^)	1.02 [1.01–1.03]	<.001
Glomerular filtration rate (GFR), ml/min/1.73 m^2^	1.00 [.99–1.01]	.118
C reactive protein (CRP), mg/L	1.00 [.99–1.01]	.148
HR bpm	1.01 [1.00–1.02]	<.001
Mean BP mm Hg	1.02 [1.01–1.03]	<.001
Age, years	1.05 [1.04–1.06]	<.001
Testosterone, nmol/L	1.00 [.99–1.01]	.651
Insulin like Growth Factor (IGF), nmol/L	1.00 [.99–1.01]	.599
HDL cholesterol, mmol/L	.85 [.80–.90]	<.001
Glucose, mmol/L	.98 [.96–1.00]	.041
Total cholesterol, mmol/L	1.16 [1.02–1.33]	.026
Triglycerides, mmol/L	1.14 [1.10–1.18]	<.001
Current tobacco (yes)	1.78 [1.67–1.91]	<.001
CV diseases (yes)	1.07 [.97–1.19]	.189
Hormone therapy (yes)	1.08 [1.01–1.17]	.020
Creatinine, micromol/L	1.00 [.99–1.01]	.333
LDL cholesterol, mmol/L	.84 [.72–.98]	.028
Uric acid lowering therapy	.80 [.53–1.19]	.233
Urea, mmol/L	1.04 [1.02–1.05]	<.001
Serum Uric Acid (SUA), mmol/L		<.001
°Q1	Ref.	
°Q2	1.05 [.99–1.10]	.101
°Q3	1.13 [1.07–1.19]	<.001
°Q4	1.22 [1.15–1.30]	<.001

Overall study population		
Parameters	OR 95%CI	P value
BMI (kg/m^2^)	1.02 [1.01–1.03]	<.001
Glomerular filtration rate (GFR), ml/min/1.73 m^2^	1.00 [.99–1.01]	.075
C reactive protein (CRP), mg/L	1.00 [.99–1.01]	.951
HR bpm	1.01 [1.00–1.02]	<.001
Mean BP mm Hg	1.02 [1.01–1.03]	<.001
Age, years	1.05 [1.04–1.06]	<.001
Testosterone, nmol/L	1.00 [.99–1.01]	.198
Insulin like Growth Factor (IGF), nmol/L	.99 [.98–1.00]	.007
HDL cholesterol, mmol/L	.73 [.66–.80]	<.001
Glucose, mmol/L	.98 [.97–.99]	<.001
Total cholesterol, mmol/L	1.08 [.99–1.18]	.082
Triglycerides, mmol/L	1.08 [1.06–1.11]	<.001
Current tobacco (yes)	1.70 [1.62–1.78]	<.001
CV diseases (yes)	1.08 [1.03–1.14]	.003
Creatinine, micromol/L	.99 [.98–1.00]	.010
LDL cholesterol, mmol/L	.93 [.84–1.03]	.176
Uric acid lowering therapy	.90 [.81–.99]	.035
Urea, mmol/L	1.02 [1.01–1.03]	.003
Serum Uric Acid (SUA), mmol/L		<.001
°Q1	Ref.	
°Q2	1.04 [1.00–1.08]	.032
°Q3	1.10 [1.06–1.14]	<.001
°Q4	1.14 [1.10–1.19]	<.001
Sex (male)	1.68 [1.57–1.79]	<.001

In the multivariate analysis among women, after adjustment for confounding factors, SUA remained an independent factor of arterial stiffness (defined by ASI > 10 m/s), with an increase in the strength of the association between the quartiles, that is, Q4 versus Q1, OR = 1.22 [1.15–1.30], *P* < .001, Q3 versus Q1, OR = 1.13 [1.07–1.19], *P* < .001 and no difference between Q2 and Q1 (*P* = .101). In the multivariate analysis, BMI (*P* < .001), HR (*P* < .001), Mean BP (*P* < .001), age (*P* < .001), HDL cholesterol (*P* < .001), total cholesterol (*P* = .026), LDL cholesterol (*P* = .028), tobacco status (*P* < .001), triglycerides (*P* < .001), fasting glucose (*P* = .041), urea (*P* < .001), and hormone therapy (*P* = .020) remained significant (**Table** **2**). When applying continuous SUA values in the multivariate analysis, SUA remained significant (*P* < .001), with a Youden index value equal to 267.3 mmol/L.

In the multivariate analysis in the overall population, after adjustment for confounding factors including sex, SUA remained an independent factor of arterial stiffness (defined by ASI > 10 m/s), with an increase in the strength of the association between the quartiles, that is, Q4 versus Q1, OR = 1.14 [1.10–1.19], *P* < .001, Q3 versus Q1, OR = 1.10 [1.06–1.14], *P* < .001 and Q2 versus Q1 OR = 1.04 [1.00–1.08], *P* = .032). In the multivariate analysis, sex (*P* < .001) BMI (*P* < .001), creatinine (*P* = .010), HR (*P* < .001), mean BP (*P* < .001), age (*P* < .001), HDL cholesterol (*P* < .001), fasting glucose (*P* < .001), tobacco status (*P* < .001), IGF (*P* = .007), triglycerides (*P* < .001), CV diseases (*P* = .003), creatinine (*P* = .010), urea (*P* = .003), and uric acid lowering therapy (*P* = .035) remained significant (**Table** **2**). When applying continuous SUA values in the multivariate analysis, SUA remained significant (*P* < .001), with a Youden index value equal to 302.0 mmol/L.

### 3.1 Sensitivity analysis

By divided the overall population according the presence of CV disease or CV risk factors (ie, hypertension and diabetes), 61 453 (48.5%) participants showed no CV diseases or CV risk factors while 65 210 (51.5%) had at least one CV disease, hypertension or diabetes. Same results were observed in men, women and in overall population regardless the status of CV diseases as observed in primary analyses (**Table** **3**).

**TABLE 3:** Multivariate logistic analyses for arterial stiffness (ASI > 10 m/s) and quartiles of SUA levels for men, women and overall study population according to the presence or not of at least one CV disease, diabetes or hypertension

MEN							
With CVD (*N* = 39 295)			.047	Without CVD (*N* = 24 797)			<.001
Q1	Ref.			Q1	Ref.		
Q2	1.02	[.96–1.08]	9.557	Q2	1.07	[.99–1.15]	.061
Q3	1.08	[1.01–1.14]	.019	Q3	1.12	[1.04–1.21]	.003
Q4	1.07	[1.01–1.13]	.041	Q4	1.19	[1.09–1.30]	<.001

WOMEN							
With CVD (*N* = 25 915)			<.001	Without CVD (*N* = 36 656)			<.001
Q1	Ref.			Q1	Ref.		
Q2	1.00	[.91–1.09]	.966	Q2	1.07	[.99–1.14]	.056
Q3	1.08	[1.01–1.18]	.048	Q3	1.16	[1.08–1.24]	<.001
Q4	1.20	[1.10–1.31]	<.001	Q4	1.26	[1.16–1.38]	<.001

Overall population							
With CVD (*N* = 65 210)			<.001	Without CVD (*N* = 61 453)			<.001
Q1	Ref.			Q1	Ref.		
Q2	1.01	[.96–1.01]	.708	Q2	1.07	[1.01–1.12]	.011
Q3	1.07	[1.02–1.13]	.004	Q3	1.13	[1.08–1.19]	<.001
Q4	1.11	[1.06–1.17]	<.001	Q4	1.21	[1.14–1.28]	<.001

CVD: cardiovascular disease (including at least heart attack, angina, stroke, diabetes or hypertension).

## 4 DISCUSSION

This cross‐sectional study investigated the association between SUA and ASI level in a large population of middle‐aged participants. A positive association between SUA quartiles and ASI was shown in multivariate analyses in both sex. These findings may suggest that higher SUA levels can possess an unfavorable action on arterial stiffness among general populations. In parallel, in our study we found the usual determinants of arterial stiffness, as age, sex, mean blood pressure and heart rate,^35^ BMI,^35^, ^36^, ^37^ tobacco status,^38^ HDL cholesterol, fasting glucose, total cholesterol, triglycerides and IGF‐1, and CV diseases.^35^, ^39^ Youden indexes were performed to discriminate arterial stiffness, with a value of SUA equal to 302.0 mmol/L in the overall population, 338.2 mmol/L in men and 267.3 mmol/L in women.

### 4.1 Cross‐sectional investigations

The relationship between SUA and arterial stiffness has been evaluated in cross‐sectional and prospective large population studies. 4140 volunteers were included in the Framingham Heart Study and presented low risk factors.^33^ In this study, SUA was associated with increased arterial stiffness, measured by PWV. This association remains significant after adjustment for confounding factors, as age, sex, hypertension, BMI, fasting plasma glucose, insulin, animal protein intake and renal function. But, the strength of this association was decreased after these adjustments, this can suggest that the relationship between SUA and arterial stiffness could be not clear among healthy participants. Moreover, a positive linear association was observed between SUA and baPWV levels in both sexs in the Korean Multi‐Rural Communities Cohort study.^40^

In contrast, other studies have shown a sex difference for this relationship, as a positive correlation in women but not in men. This has been observed among 66 917 middle‐aged Koreans with low CV risk factors, and where men present a J‐shaped correlation. The conclusions of the authors were that SUA may present an unfavorable vascular effects among low‐risk population but the ethnic lifestyle could influence this relationship.^14^

### 4.2 Sex differences

In our study we have shown an age‐interaction with SUA for arterial stiffness in both sex. A sex difference has been observed in different studies for SUA levels.^41^ Women presented lower SUA levels than men. Aging according to sex may also influence this association, where healthy older women with hyperuricemia showed increased arterial stiffness risk.^42^ The sex difference for this relationship could be due to the difference on prevalence of current smokers, hypertension, age, dyslipidemia between men and sex.^43^ Furthermore, the association between SUA levels and CV events is stronger in women than in men.^44^ Metabolic syndrome was closely related to SUA levels in women than in men.^45^ These observation are consistent to the results of our study where women presented higher OR than men for the relationship between SUA levels and AS (**Table** **2**). A meta‐analysis showed that the relationship between CV mortality and SUA levels was significant in women but not in men.^46^ Nevertheless, few studies have clearly investigated the association between SUA levels and arterial stiffness. However, a sex‐specific association could be highlighted among women between SUA and arteriosclerosis.^47^

An inverse relationship between SUA and estrogen has been observed in both sex.^48^, ^49^ Estrogen has a direct effects on sex‐associated UA transporters, such as ABCG2.^50^ After menopause, the decrease in sex hormonal levels, as estrogen, induces increased SUA levels and this decrease can be mitigated by hormonal replacement therapy.^51^ However, even if few women reported to have hormone medication, most women in the study were probably menopausal. Thus, the decrease in estrogen may have a greater effect in women than in men who per se low in estrogen. This could explain the higher relationship between SUA and arterial stiffness in women than in men. In our study, the relationship between SUA and AS remained significant after adjustment including hormone medication showing that estrogen could not be the only explained pathway in women.

### 4.3 Prospective investigations

During a follow‐up period of 6 years, repeated measures of SUA and cfPWV were performed and showed that a significant and independently association was observed over time in men but not in women.^52^ In this study, the sex differences was characterized by the fact that men had higher values of SUA than women. A longer exposure to high levels of SUA could be associated with increased risk of vascular stiffness.^52^ Among normotensive participants with a 1‐year follow‐up, SUA levels was associated with increased baPWV levels after adjustment for age, sex, BMI, lipid parameters and systolic BP.^53^

### 4.4 Pathophysiological mechanism of SUA on arterial stiffness

Two main mechanisms can explain the relationship between arterial stiffness and hyperuricemia. The independent urate crystal is characterized by the translocation of soluble SUA into the vascular wall through Glut‐9 (a glucose transporter) or via URATv1 (a voltage‐driven urate efflux transporter‐1) to induce an intra‐cellular inflammation and then, oxidative stress.^54^

The urate crystal‐dependent mechanism, activated by vascular macrophages, lead to the activation of NLRP3 (nod‐like receptor family proteins 3) inflammasome. This inflammasome cleaves pro‐IL‐1beta into activated IL‐1beta to enhance inflammation and the formation of collagen.^55^ Nevertheless this process should be only considered among patients with high UA levels, due to the crystallized UA starting level within the body when UA level exceeds its solubility limit (6.8 mg/dl).^56^

Moreover, hyperuricemia downregulates the production of NO (nitric oxide) in endothelial cells,^57^ this, in association with the activation of the renin‐angiotensin‐aldosterone (RAA) system by high UA levels.^58^ The downregulation of the bioavailability and production in NO leads to the dysfunction of endothelial cells, to increase vascular tone and then, to arterial stiffness^59^ In parallel, the RAA‐activated by hyperuricemia is responsible for activation of the different processes of arterial stiffness in cytoskeleton of endothelial cells^60^ and fibrosis of extracellular matrix.^61^ Oxidative stress induced by UA stimulates the production of endothelin‐1^62^ which is a vasoconstrictor and enhancer of arterial stiffness.^63^

The proliferation of vascular smooth muscle cells (VSMC) was induced by high UA levels by stimulating the inflammation, via Protein‐1,^64^ COX‐2^65^ and NF‐kB pathway responsible for proatherogenic effects in the vascular wall.^66^ Moreover, CRP was stimulated by UA, as observed with the proinflammatory marker, TNF‐α.^67^ CRP stimulates the production of cellular adhesion molecules, enhances cellular apoptosis and leads to endothelial dysfunction and then, arterial stiffness.^68^

SUA is the end product of purine metabolism, mist of which is excreted in the urine by kidneys.^69^ Studies have observed that increased levels of SUA were mainly associated with CKD risk onset.^70^ Moreover, a recent study showed that hyperuricemia could be considered as an independent risk factor for the development of new‐onset CKD.^71^ UA produces reactive oxygen species and angiotensin II which contribute to vascular endothelial cell aging and initiation of atherosclerosis.^72^ The increase in SUA can promote inflammation, oxidative stress, higher level of renin‐angiotensin system activity and reduction in GFR by increased renal vascular resistance due to endothelial cell damages.^72^

### 4.5 Limitations

The main strength of this study is the very large sample size of the cohort. The cross‐sectional observational design limits the relationship of causality. Reverse causation can't be ruled out. A potential limitation could stem from the utilization of Pulse Trace device to measure arterial stiffness on account of greater variability in ASI values relative to other available devices.^73^ The UK Biobank study showed a low response rate of 5.5% and possible volunteers bias may be involved. Nevertheless, given the large sample size and high internal validity, these are unlikely to affect the reported associations.^74^, ^75^ In addition, the study cohort consisted of middle‐aged European participants, so our findings may not be generalized to other age groups and ethnic populations. Uric acid lowering therapy could be underestimated in the study. Only five medications were reported, as allopurinol, benzbromarone, febuxostat, probenecid or sulfinpyrazone, according to other uric acid studies in UK Biobank.^29^, ^76^ This underestimation could be a bias in the association between SUA and arterial stiffness. Menopausal women proportion and estrogen levels were not reported in the study. The possible high proportion of menopausal women in the study and the low estrogen level in women could be a response of the higher relationship between women and SUA than men.

## 5 CONCLUSIONS

In conclusions, SUA levels appeared to be independent determinants of arterial stiffness, defined by ASI > 10 m/s, in both sex of a general middle‐aged population. Few studies have shown the significant relationship between ASI and SUA in a general population, and future prospective cohort studies are needed to better explore this link.

## CONFLICT OF INTEREST

The author declares no conflict of interest.

## AUTHOR CONTRIBUTIONS

Conceptualization, Alexandre Vallée; methodology, Alexandre Vallée; formal analysis, Alexandre Vallée; writing—original draft preparation, Alexandre Vallée; The author has read and agreed to the published version of the manuscript.
